# Secretory Carcinoma of the Breast: A Rare Entity With Favorable Prognosis

**DOI:** 10.7759/cureus.60430

**Published:** 2024-05-16

**Authors:** Souad Margoum, Soufiane Berhili, Meriem Bouabid, Mohamed Moukhlissi, Mezouar Loubna

**Affiliations:** 1 Radiation Oncology, Faculty of Medicine and Pharmacy, Mohammed First University, Oujda, MAR

**Keywords:** breast cancer, case report, treatment, prognosis, secretory carcinoma of the breast, breast

## Abstract

Secretory breast carcinoma (SBC) is an extremely rare entity of breast cancer, which can affect all age groups. The diagnosis is based on the characteristic microscopic appearance, and despite the triple negativity or low hormone receptor positivity, SBC is generally characterized by a favorable prognosis.

Due to the rarity of the disease, no clear consensus on optimal treatment is available. Nevertheless, conservative surgery or mastectomy is the main therapeutic option. The efficacy of chemotherapy, radiotherapy, and hormone therapy in this pathology has not been rigorously explored. We report the case of a 65-year-old woman with SBC treated with surgery and adjuvant radiotherapy, with a review of the literature.

## Introduction

Secretory breast carcinoma (SBC) is an extremely rare entity, representing less than 0.15% of invasive breast cancers [1]. It can affect all age groups, from 3 to 86 years, with two principal peaks in incidence: in adolescents, on average at 17 years, and in adults at around 56 years. It affects both men and women, with a sex ratio ranging from ½ to 1/6 [2,3].

Mammary secretory carcinoma is characterized by distinctive histopathological and molecular properties, a specific translocation t (12; 15) (p13: q25) responsible for an ETV6-NTRK3 fusion, a slow evolution, and a favorable prognosis [4,5].

The etiology of SBC is unknown, and no particular risk factors have been pinpointed at present. However, previous studies have examined the correlation between certain risk factors and the overall incidence of breast cancer, including secretory carcinoma of the breast. These studies have established that smoking and obesity represent significant risk factors for the development of breast cancer overall, but further research is needed to determine their specific effects in the context of SBC because of the unique characteristics of this type of cancer and its intrinsic rarity. Also, several cases of SBC have been found in women who have a history of radiation exposure or breast surgery, although conclusive evidence of these cases is still inadequate [6].

In this study, we present the case of a 65-year-old woman with SBC, successfully managed by surgery and adjuvant radiotherapy, with a review of the literature

## Case presentation

The patient was a 65-year-old woman, menopausal, G0P0, with no personal or family history of breast cancer. She was admitted for a painless mass in the right breast, with no inflammatory signs neither skin retraction nor nipple discharge, detected at breast self-exam five months earlier.

Physical examination revealed a 3.5 cm mass at the junction of the superior external quadrant and retro-areolar area of the right breast. The mass had a hard consistency and was associated with a unique mobile ipsilateral axillary lymph node measuring 1.5 cm.

**Figure 1:**
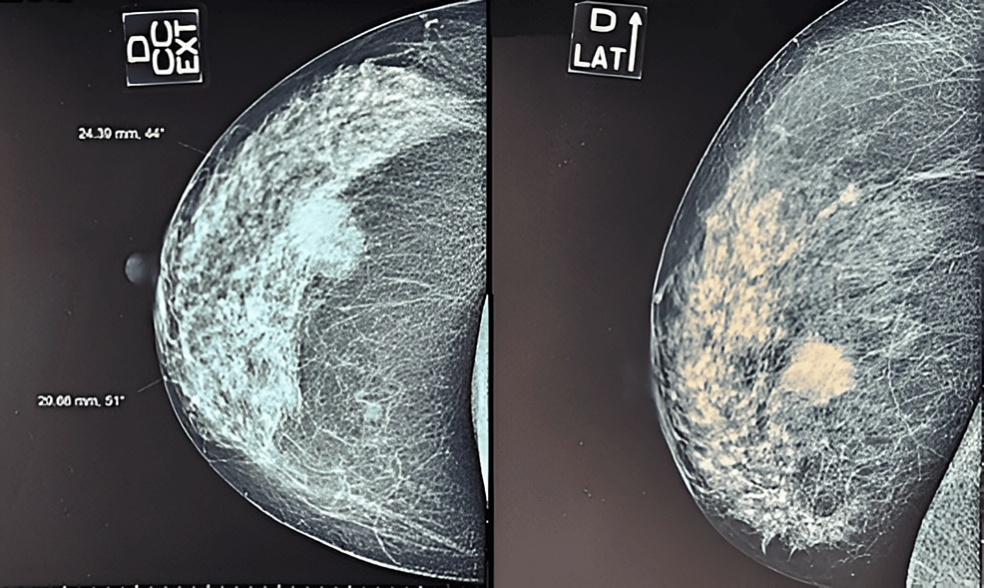
Mammography image showing a rounded mass with irregular contours at the junction of the outer quadrants of the right breast

Bilateral mammography showed a rounded mass with irregular contours at the junction of the outer quadrants of the right breast, measuring 29*24 mm, as well as a 6 mm opacity in the superior-external quadrant (Figure 1).

The ultrasound confirmed the presence of an irregularly contoured, hypoechoic, heterogeneous nodule measuring 23*22*21 mm at the junction of the external quadrants of the right breast. A 11*7mm adenomegaly of the right axillary extension was also detected. These abnormalities were classified as BIRADS 5 type of ACR classification.

Tru-Cut biopsy was performed, revealing a proliferation of carcinoma cells arranged in solid lobules, thin trabeculae, and tubular-glandular formations, initially interpreted as an SBR grade 2 infiltrating ductal carcinoma.

Thoraco-abdomen-pelvic CT scan did not find any lesion suspicious of distant metastasis.

The patient underwent right mastectomy with ipsilateral axillary lymphadenectomy. Histological evaluation showed an infiltrating secretory-type breast carcinoma, measuring 22 mm long axis, SBR grade 2. No vascular emboli or peri-nervous sheathing was observed.

**Figure 2:**
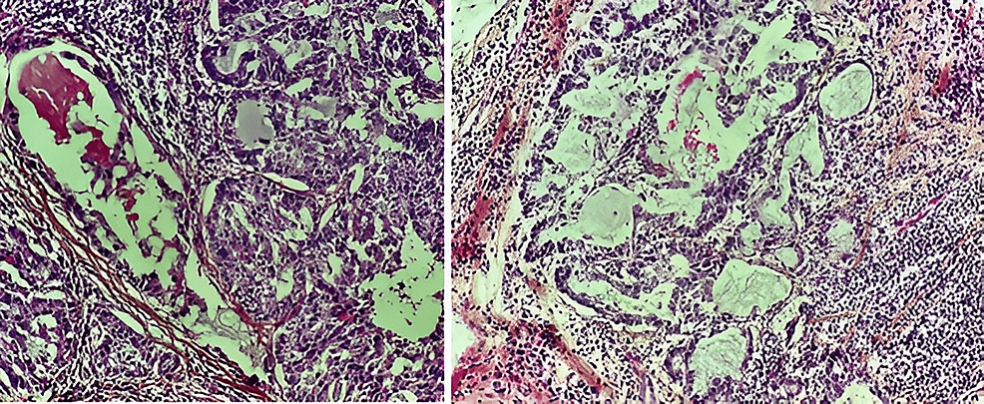
Histological image showing tumor proliferation arranged in cribriform masses and glands whose lumens are filled with secretions, occasionally eosinophilic (HE, x 20)

**Figure 3:**
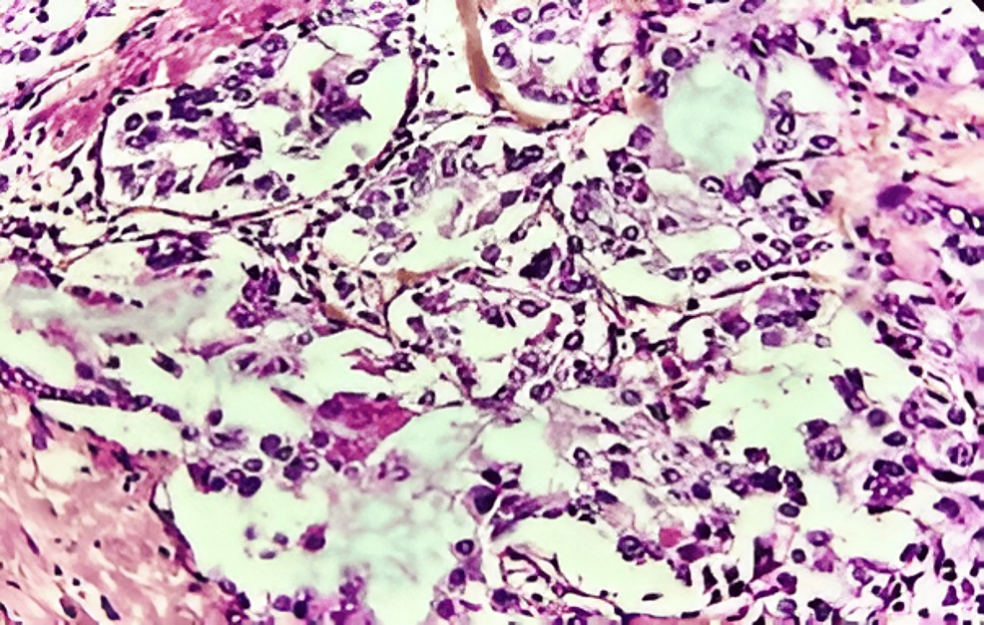
Tumor cells show moderate cytonuclear atypia, with a vesicular nucleus and eosinophilic cytoplasm (HE, x 40)

An in situ component of cribriform and solid architecture of high nuclear grade, with multifocal comedo necrosis was present in 10% of the tumor. Tumor-infiltrating lymphocytes were estimated at 60%. The nipple and skin were not infiltrated, and there was no Paget's disease. Twelve non-metastatic lymph nodes were found at the axillary lymph node dissection (Figures 2, 3).

Immunohistochemical analysis showed negative staining for estrogen and progesterone hormone receptors, and HER2. The Ki67 proliferation index was estimated at 50%. Thus, the tumor was classified pT2N0M0 / Triple negative subtype. 

At the Pluridisciplinary Concertation Meeting, an adjuvant radiotherapy was indicated without adjuvant chemotherapy. The patient received external hypofractionated 3D conformal radiotherapy to the right chest wall, at a total dose of 42 Gy in 15 fractions of 2.8 Gy over 18 days, with a good clinical tolerance.

After six months, the patient was regularly followed-up, with no clinical or radiological evidence of disease relapse.

## Discussion

The first description of SBC dates back to 1966 by McDivitt and Stewart, who observed seven cases of "juvenile breast cancer" in girls aged between 3 and 15 years old, presenting with similar clinical and histological symptoms [1]. Subsequently, Tavassoli and Norris described the abundant secretion of eosinophils in intracellular vacuoles and intercellular spaces, introducing the term "secretory carcinoma" [7]. Currently, the majority of published cases (around two-thirds) of SBCs in the literature describe the adult population, with an average age of 56 [3]

Generally, most patients with secretory breast cancer experience clinical manifestations at an early stage, with a relatively slow clinical progression. Approximately 30% of cases present with lymph node metastases, while distant metastases remain rare [8]. The SBC usually manifests as a firm, painless mass, mobile to palpation, with a predominant location in the upper outer quadrant of the breast. It is rarely multicentric, and some SBC patients may present with a nipple discharge [9,10]. Tumor size in SBC is a prognostic indicator. Indeed, a median size of 5.3 cm has been reported in locally advanced tumors, exceeding the dimensions typically observed in other breast cancer subtypes [11]. This particularity may explain the frequent initial confusion of SBC with benign lesions.

Mammography remains of limited diagnostic value in SBC, as it usually presents as a single, discrete mass, lobular, with smooth or irregular edges. These characteristics may resemble a fibroadenoma more strongly than a malignant tumor. The distinctive ultrasound appearance of SBC reveals an oval, round, and tubular mass with hypoechoic or isoechoic echogenicity. In general, it keeps a benign appearance, manifesting itself either as a single mass or as a cluster of nodules [12]. Magnetic resonance imaging (MRI) also has diagnostic value, although only in exceptional cases T2-weighted sequences can show a complex cystic mass, with early enhancement after injection of gadolinium [5].

The diagnosis of SBC is based essentially on its characteristic microscopic appearance, revealing cells with vacuolated cytoplasm and abundant intra- and extracellular milk-like eosinophilic secretions [10]. These SBC features are often hard to differentiate from those of invasive ductal carcinoma, notably with the presence of both types of tubular structures and desmoplastic tumor stroma with blood vessel elastosis [5]. Positive PAS (Schiff's periodic acid) reactivity of secretions represents a distinctive diagnostic feature of SBC [2]. In our case, there was no need to perform PAS staining in an immunohistochemical study as the abundance of extracellular secretion allowed the confirmation of the diagnosis. There are primarily three histological presentations observed, namely the solid, microcystic, tubular presentation, or a combination of the three, and rarely papillary [10,13]. Tumor cells are typically low-grade, characterized by small to medium-sized, oval-to-round nuclei, low mitotic activity, and abundant granular eosinophilic cytoplasm [2]. However, our case has a high proliferation index (Ki67=50%), which has been in favor of the indication of adjuvant radiotherapy.

Various studies have established that SBC is generally characterized by triple negativity or low positivity for hormone receptors, in association with positivity for S-100 protein. However, more recent studies have called into question the specificity of S-100 protein expression in SBC [4,14]. In addition, further research has identified cases with moderate to high estrogen receptor expression [4].

With the development of molecular detection technologies, the pan-TRK protein has become a sensitive and specific marker for ETV6-NTRK fusion tumors, targeting a conserved sequence near the C-terminus of TRK proteins. The scientific literature suggests that the distinct morphology of the tumor, combined with diffuse and intense nuclear staining, at least focally, can serve as an initial diagnostic criterion in most cases [13,14]. Fluorescence in situ hybridization (FISH) is still recommended for patients with atypical histomorphology or lack of pan-TRK protein expression [5,14]. There was no need for such investigations in our case as the diagnosis was evident in pathology examination.

Due to its rarity, SBC has no clear consensual treatment. Management approaches vary and are impacted by factors such as the patient's age and preferences, hormone positivity, tumor size, and lymph node involvement [5]. Total or partial mastectomy remains the principal therapeutic modality, but the necessity and extent of axillary lymph node dissection is debatable, depending on the specific clinical presentation, due to the tumor's limited metastatic potential [8,11]. Adjuvant radiotherapy is indicated in patients who have undergone conservative surgery, although evidence of its definitive benefit in SBC is still limited [2,6,8]. Similarly, the efficacy of chemotherapy in this pathology has not been rigorously studied [6]. Our patient received only adjuvant radiotherapy with no adjuvant chemotherapy, given the lack of efficiency of the latter. Hormone therapy has also proved to be effective in certain cases, especially in patients expressing positive estrogen or progesterone receptor staining [6], which wasn’t the case in our patient.

SBC has generally a good prognosis, with a five-year survival rate exceeding 95% [6]. However, some cases may experience local recurrence, a more aggressive clinical evolution, with distant metastases and poorer results, particularly when it presents at an inflammatory stage before initial treatment; Therefore, a follow-up period reaching 20 years is recommended [10,15].

## Conclusions

SBC is a very rare and heterogeneous subtype of breast cancer characterized by an indolent clinical evolution, a low incidence of systemic metastases, and an excellent prognosis. Diagnosis remains complex, particularly in elderly patients, and often requires complementary methods such as immunohistochemical studies and FISH. Surgical resection with clear margins remains the cornerstone of treatment, sometimes followed by adjuvant radiotherapy. However, due to the rarity of this disease and the diversity of its clinical presentations, further research is needed to establish optimal, well-standardized management strategies for patients with SBC.
